# 1,1′-Bis[bis­(4-meth­oxy­phen­yl)phosphan­yl]ferrocene

**DOI:** 10.1107/S1600536812026293

**Published:** 2012-06-16

**Authors:** Xinfeng Ren, Le Wang, Ya Li

**Affiliations:** a College of Chemistry and Chemical Engineering, Shanghai University of Engineering Science, Shanghai 201620, People’s Republic of China

## Abstract

In the crystal structure of the title substituted ferrocene complex, [Fe(C_19_H_18_O_2_P)_2_], the Fe^II^ atom lies on a twofold rotation axis, giving an eclipsed cyclo­penta­dienyl conformation with a ring centroid separation of 3.292 (7) Å and an Fe—C bond-length range of 2.0239 (15)–2.0521 (15) Å. In the ligand, the cyclo­penta­dienyl ring forms dihedral angles of 60.36 (6) and 82.93 (6)° with the two benzene rings of the diphenyl­phosphine group, while the dihedral angle between the benzene rings is 67.4 (5)°.

## Related literature

For the synthesis of the title compound from ferrocene, see: Ogasawara *et al.* (2002 ▶). For applications of the title compound, see: Gusev *et al.* (2006 ▶); Hamann & Hartwig (1998 ▶); Casellato *et al.* (1988 ▶).
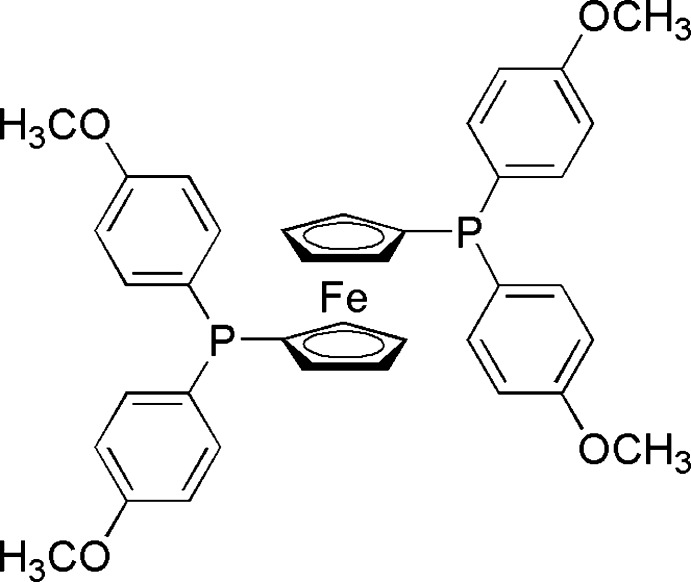


## Experimental

### 

#### Crystal data

[Fe(C_19_H_18_O_2_P)_2_]
*M* *_r_* = 674.46
Monoclinic, 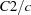

*a* = 19.0790 (8) Å
*b* = 9.9445 (4) Å
*c* = 17.5663 (8) Å
β = 102.386 (1)°
*V* = 3255.3 (2) Å^3^
*Z* = 4
Mo *K*α radiation
μ = 0.60 mm^−1^
*T* = 173 K
0.48 × 0.46 × 0.32 mm

#### Data collection

Bruker SMART CCD area-detector diffractometer
Absorption correction: multi-scan (*SADABS*; Sheldrick, 1996 ▶) *T*_min_ = 0.761, *T*_max_ = 0.831
18254 measured reflections
2869 independent reflections
2740 reflections with *I* > 2σ(*I*)
*R*_int_ = 0.019

#### Refinement

*R*[*F* ^2^ > 2σ(*F* ^2^)] = 0.026
*wR*(*F* ^2^) = 0.070
*S* = 1.04
2869 reflections
204 parameters
H-atom parameters constrained
Δρ_max_ = 0.34 e Å^−3^
Δρ_min_ = −0.18 e Å^−3^

### 

Data collection: *SMART* (Bruker, 2007 ▶); cell refinement: *SAINT* (Bruker, 2007 ▶); data reduction: *SAINT*; program(s) used to solve structure: *SHELXS97* (Sheldrick, 2008 ▶); program(s) used to refine structure: *SHELXL97* (Sheldrick, 2008 ▶); molecular graphics: *SHELXTL* (Sheldrick, 2008 ▶); software used to prepare material for publication: *SHELXTL*.

## Supplementary Material

Crystal structure: contains datablock(s) I, global. DOI: 10.1107/S1600536812026293/zs2202sup1.cif

Supplementary material file. DOI: 10.1107/S1600536812026293/zs2202Isup2.cdx

Structure factors: contains datablock(s) I. DOI: 10.1107/S1600536812026293/zs2202Isup3.hkl

Supplementary material file. DOI: 10.1107/S1600536812026293/zs2202Isup4.cdx

Additional supplementary materials:  crystallographic information; 3D view; checkCIF report

## Figures and Tables

**Fig. 1.**
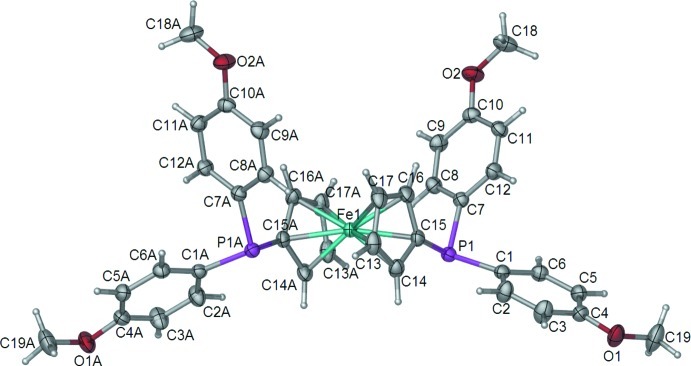
Molecular configuration and atom-numbering scheme for the title compound, with probability displacement ellipsoids drawn at the 50% level. For symmetry code (i): -x, y, -z+1/2.

## supplementary crystallographic information

### Comment

The title compound is a substituted ferrocene complex [C_38_H_36_FeO_4_P_2_] which was synthesized in a reaction of 4-methoxyphenylmagnesium bromide with 1,1'-bis(dichlorophosphine)ferrocene. A previously reported synthesis involved the reaction of chlorobis(4-methoxyphenyl)phosphine with ferrocene (Ogasawara *et al.*, 2002). A potential application of the compound is in metal-catalized organic reactions (Gusev *et al.*, 2006; Hamann & Hartwig, 1998). The single-crystal structure determination of the compound was performed to provide the coordination geometry and structural conformation, which will allow the study of the mechanism of metal-catalized organic reactions.

In the structure of the title compound, the Fe^II^ lies on a twofold rotation axis giving an eclipsed cyclopentadienyl configuration (Fig. 1), with a cyclopentadienyl ring-centroid separation of 3.292 (7) Å and an Fe—C range of 2.0239 (15)–2.0521 (15) Å. These distances compare with 3.305 (3) and 2.033 (4)–2.064 (4) Å in a similar ferrocene complex (Casellato *et al.*, 1988). In each ligand, the cyclopentadienyl ring forms dihedral angles of 60.36 (6)° and 82.93 (6)° with the two phenyl rings of the diphenylphosphine substituent group, while the dihedral angle between the phenyl rings is 67.4 (5)°.

### Experimental

A solution of 4-methoxyphenylmagnesium bromide (1 *M* in THF, 12 ml, 12 mmol) was added dropwise over 5 min. at -78 °C to a stirred solution of 1,1'-bis(dichlorophosphanyl)ferrocene (776 mg, 2 mmol) in THF (15 ml) under argon. The mixture was warmed to 25 °C slowly and was stirred for an additional 12 h. The reaction was monitored by PNMR. The resulting mixture was quenched with water (2 ml) at 0 °C, and then filtered and washed with water (10×, 3 ml), methanol (5×, 2 ml) and diethyl ether (5×, 3 ml). The solid obtained was then dissolved in chloroform (5 ml) and the solution was passed through a short silica gel plug, and washed with chloroform (5×, 3 ml) to remove the residual salt. The solvent was removed and the resulting solid was dried under vacuum to give the title compound as a yellow solid (680 mg, 50.5% yield). Single crystals suitable for X-ray diffraction are obtained from a CH_2_Cl_2_-hexane solution *via* solvent evaporation at room temperature after two weeks.

### Refinement

All H-atoms were placed in calculated positions with C—H = 0.93 Å and were allowed to ride in the refinement, with U</>_iso_(H) = 1.2U_eq_(C).

### Crystal data

**Table:**

[Fe(C_19_H_18_O_2_P)_2_]	*F*(000) = 1408
*M**_r_* = 674.46	*D*_x_ = 1.376 Mg m^−^^3^
Monoclinic, *C*2/*c*	Mo *K*α radiation, λ = 0.71073 Å
Hall symbol: -C 2yc	Cell parameters from 9910 reflections
*a* = 19.0790 (8) Å	θ = 2.3–28.3°
*b* = 9.9445 (4) Å	µ = 0.60 mm^−^^1^
*c* = 17.5663 (8) Å	*T* = 173 K
β = 102.386 (1)°	Block, yellow
*V* = 3255.3 (2) Å^3^	0.48 × 0.46 × 0.32 mm
*Z* = 4	

### Data collection

**Table:**

Bruker SMART CCD area-detector diffractometer	2869 independent reflections
Radiation source: fine-focus sealed tube	2740 reflections with *I* > 2σ(*I*)
Graphite monochromator	*R*_int_ = 0.019
φ and ω scans	θ_max_ = 25.0°, θ_min_ = 2.2°
Absorption correction: multi-scan (*SADABS*; Sheldrick, 1996)	*h* = −22→22
*T*_min_ = 0.761, *T*_max_ = 0.831	*k* = −11→11
18254 measured reflections	*l* = −20→20

### Refinement

**Table:**

Refinement on *F*^2^	Primary atom site location: structure-invariant direct methods
Least-squares matrix: full	Secondary atom site location: difference Fourier map
*R*[*F*^2^ > 2σ(*F*^2^)] = 0.026	Hydrogen site location: inferred from neighbouring sites
*wR*(*F*^2^) = 0.070	H-atom parameters constrained
*S* = 1.04	*w* = 1/[σ^2^(*F*_o_^2^) + (0.0343*P*)^2^ + 3.7311*P*] where *P* = (*F*_o_^2^ + 2*F*_c_^2^)/3
2869 reflections	(Δ/σ)_max_ < 0.001
204 parameters	Δρ_max_ = 0.34 e Å^−^^3^
0 restraints	Δρ_min_ = −0.18 e Å^−^^3^

### Special details

**Table:**

Geometry. All e.s.d.'s (except the e.s.d. in the dihedral angle between two l.s. planes) are estimated using the full covariance matrix. The cell e.s.d.'s are taken into account individually in the estimation of e.s.d.'s in distances, angles and torsion angles; correlations between e.s.d.'s in cell parameters are only used when they are defined by crystal symmetry. An approximate (isotropic) treatment of cell e.s.d.'s is used for estimating e.s.d.'s involving l.s. planes.
Refinement. Refinement of *F*^2^ against ALL reflections. The weighted *R*-factor *wR* and goodness of fit *S* are based on *F*^2^, conventional *R*-factors *R* are based on *F*, with *F* set to zero for negative *F*^2^. The threshold expression of *F*^2^ > σ(*F*^2^) is used only for calculating *R*-factors(gt) *etc*. and is not relevant to the choice of reflections for refinement. *R*-factors based on *F*^2^ are statistically about twice as large as those based on *F*, and *R*- factors based on ALL data will be even larger.

### Fractional atomic coordinates and isotropic or equivalent isotropic displacement parameters (Å^2^)

**Table:**

	*x*	*y*	*z*	*U*_iso_*/*U*_eq_	
Fe1	0.0000	0.11062 (3)	0.2500	0.02319 (10)	
P1	0.11216 (2)	0.17867 (4)	0.12853 (2)	0.02453 (11)	
O1	0.06477 (7)	0.44156 (13)	−0.18422 (7)	0.0424 (3)	
O2	0.22904 (6)	−0.37992 (11)	0.13592 (7)	0.0382 (3)	
C1	0.09423 (8)	0.23741 (15)	0.02723 (9)	0.0248 (3)	
C2	0.03081 (9)	0.30448 (18)	−0.00579 (10)	0.0366 (4)	
H2A	−0.0076	0.3054	0.0209	0.044*	
C3	0.02282 (10)	0.3694 (2)	−0.07642 (11)	0.0414 (4)	
H3A	−0.0210	0.4140	−0.0979	0.050*	
C4	0.07794 (9)	0.37024 (16)	−0.11638 (9)	0.0302 (3)	
C5	0.14120 (8)	0.30234 (16)	−0.08555 (9)	0.0298 (3)	
H5A	0.1791	0.3003	−0.1129	0.036*	
C6	0.14846 (8)	0.23725 (16)	−0.01420 (9)	0.0287 (3)	
H6A	0.1919	0.1912	0.0068	0.034*	
C7	0.14856 (8)	0.00986 (15)	0.12251 (9)	0.0250 (3)	
C8	0.19314 (8)	−0.04129 (16)	0.18999 (9)	0.0280 (3)	
H8A	0.2058	0.0142	0.2349	0.034*	
C9	0.21914 (8)	−0.17124 (16)	0.19252 (9)	0.0312 (3)	
H9A	0.2488	−0.2049	0.2390	0.037*	
C10	0.20173 (8)	−0.25261 (15)	0.12684 (9)	0.0281 (3)	
C11	0.15920 (8)	−0.20285 (16)	0.05856 (9)	0.0298 (3)	
H11A	0.1484	−0.2574	0.0131	0.036*	
C12	0.13253 (8)	−0.07256 (16)	0.05713 (9)	0.0292 (3)	
H12A	0.1028	−0.0392	0.0106	0.035*	
C13	−0.08978 (9)	0.16360 (19)	0.16939 (9)	0.0356 (4)	
H13A	−0.1341	0.2053	0.1808	0.043*	
C14	−0.02860 (9)	0.23385 (17)	0.15681 (9)	0.0311 (3)	
H14A	−0.0226	0.3338	0.1572	0.037*	
C15	0.02275 (8)	0.13737 (16)	0.14232 (8)	0.0261 (3)	
C16	−0.00775 (9)	0.00717 (17)	0.14745 (9)	0.0314 (3)	
H16A	0.0158	−0.0808	0.1411	0.038*	
C17	−0.07674 (9)	0.02422 (19)	0.16437 (9)	0.0370 (4)	
H17A	−0.1102	−0.0497	0.1719	0.044*	
C18	0.21229 (11)	−0.46909 (18)	0.07098 (12)	0.0456 (4)	
H18A	0.2351	−0.5564	0.0854	0.068*	
H18B	0.2301	−0.4313	0.0272	0.068*	
H18C	0.1602	−0.4809	0.0557	0.068*	
C19	0.11777 (11)	0.4448 (3)	−0.22871 (12)	0.0557 (6)	
H19A	0.1009	0.4995	−0.2755	0.084*	
H19B	0.1275	0.3531	−0.2440	0.084*	
H19C	0.1618	0.4842	−0.1977	0.084*	

### Atomic displacement parameters (Å^2^)

**Table:**

	*U*^11^	*U*^22^	*U*^33^	*U*^12^	*U*^13^	*U*^23^
Fe1	0.02589 (17)	0.02243 (17)	0.02163 (17)	0.000	0.00595 (12)	0.000
P1	0.0268 (2)	0.0228 (2)	0.0238 (2)	−0.00004 (14)	0.00500 (15)	−0.00076 (15)
O1	0.0477 (7)	0.0485 (7)	0.0333 (6)	0.0126 (6)	0.0140 (5)	0.0159 (6)
O2	0.0407 (7)	0.0262 (6)	0.0440 (7)	0.0073 (5)	0.0007 (5)	−0.0013 (5)
C1	0.0274 (7)	0.0213 (7)	0.0253 (7)	−0.0014 (6)	0.0047 (6)	−0.0006 (6)
C2	0.0303 (8)	0.0469 (10)	0.0350 (9)	0.0070 (7)	0.0121 (7)	0.0097 (8)
C3	0.0320 (9)	0.0526 (11)	0.0402 (10)	0.0145 (8)	0.0095 (7)	0.0140 (8)
C4	0.0368 (9)	0.0263 (8)	0.0276 (8)	0.0014 (6)	0.0070 (7)	0.0026 (6)
C5	0.0307 (8)	0.0305 (8)	0.0303 (8)	0.0006 (6)	0.0114 (6)	−0.0001 (7)
C6	0.0277 (7)	0.0276 (8)	0.0305 (8)	0.0039 (6)	0.0057 (6)	0.0016 (6)
C7	0.0237 (7)	0.0251 (7)	0.0263 (7)	−0.0001 (6)	0.0058 (6)	0.0008 (6)
C8	0.0271 (7)	0.0301 (8)	0.0255 (7)	−0.0009 (6)	0.0025 (6)	−0.0027 (6)
C9	0.0292 (8)	0.0326 (9)	0.0290 (8)	0.0029 (6)	−0.0005 (6)	0.0043 (7)
C10	0.0245 (7)	0.0245 (7)	0.0357 (8)	0.0005 (6)	0.0075 (6)	0.0019 (6)
C11	0.0326 (8)	0.0289 (8)	0.0275 (8)	−0.0006 (6)	0.0050 (6)	−0.0041 (6)
C12	0.0321 (8)	0.0296 (8)	0.0238 (8)	0.0030 (7)	0.0014 (6)	0.0020 (6)
C13	0.0272 (8)	0.0549 (11)	0.0240 (8)	0.0054 (7)	0.0041 (6)	0.0032 (7)
C14	0.0344 (8)	0.0340 (9)	0.0254 (8)	0.0081 (7)	0.0075 (6)	0.0064 (7)
C15	0.0298 (8)	0.0285 (8)	0.0203 (7)	0.0008 (6)	0.0058 (6)	0.0008 (6)
C16	0.0371 (9)	0.0317 (8)	0.0261 (8)	−0.0071 (7)	0.0085 (7)	−0.0071 (6)
C17	0.0341 (9)	0.0498 (10)	0.0270 (8)	−0.0136 (8)	0.0068 (7)	−0.0084 (7)
C18	0.0499 (11)	0.0287 (9)	0.0560 (12)	0.0055 (8)	0.0067 (9)	−0.0084 (8)
C19	0.0541 (12)	0.0745 (15)	0.0435 (11)	0.0102 (11)	0.0215 (9)	0.0259 (11)

### Geometric parameters (Å, º)

**Table:**

Fe1—C14	2.0239 (15)	C6—H6A	0.9500
Fe1—C14^i^	2.0239 (15)	C7—C12	1.390 (2)
Fe1—C13^i^	2.0436 (16)	C7—C8	1.398 (2)
Fe1—C13	2.0437 (16)	C8—C9	1.381 (2)
Fe1—C15	2.0467 (14)	C8—H8A	0.9500
Fe1—C15^i^	2.0468 (14)	C9—C10	1.390 (2)
Fe1—C17	2.0493 (16)	C9—H9A	0.9500
Fe1—C17^i^	2.0493 (16)	C10—C11	1.387 (2)
Fe1—C16^i^	2.0521 (15)	C11—C12	1.390 (2)
Fe1—C16	2.0521 (15)	C11—H11A	0.9500
P1—C15	1.8207 (16)	C12—H12A	0.9500
P1—C7	1.8284 (15)	C13—C17	1.414 (3)
P1—C1	1.8341 (15)	C13—C14	1.418 (2)
O1—C4	1.3630 (19)	C13—H13A	1.0000
O1—C19	1.405 (2)	C14—C15	1.432 (2)
O2—C10	1.3653 (19)	C14—H14A	1.0000
O2—C18	1.426 (2)	C15—C16	1.430 (2)
C1—C6	1.387 (2)	C16—C17	1.420 (2)
C1—C2	1.394 (2)	C16—H16A	1.0000
C2—C3	1.378 (2)	C17—H17A	1.0000
C2—H2A	0.9500	C18—H18A	0.9800
C3—C4	1.384 (2)	C18—H18B	0.9800
C3—H3A	0.9500	C18—H18C	0.9800
C4—C5	1.388 (2)	C19—H19A	0.9800
C5—C6	1.391 (2)	C19—H19B	0.9800
C5—H5A	0.9500	C19—H19C	0.9800
			
C14—Fe1—C14^i^	105.47 (10)	C6—C5—H5A	120.4
C14—Fe1—C13^i^	116.41 (7)	C1—C6—C5	122.08 (14)
C14^i^—Fe1—C13^i^	40.79 (7)	C1—C6—H6A	119.0
C14—Fe1—C13	40.80 (7)	C5—C6—H6A	119.0
C14^i^—Fe1—C13	116.41 (7)	C12—C7—C8	118.15 (14)
C13^i^—Fe1—C13	150.12 (11)	C12—C7—P1	124.75 (12)
C14—Fe1—C15	41.20 (6)	C8—C7—P1	117.03 (11)
C14^i^—Fe1—C15	126.51 (6)	C9—C8—C7	121.10 (14)
C13^i^—Fe1—C15	107.13 (6)	C9—C8—H8A	119.5
C13—Fe1—C15	68.81 (6)	C7—C8—H8A	119.5
C14—Fe1—C15^i^	126.51 (6)	C8—C9—C10	119.85 (14)
C14^i^—Fe1—C15^i^	41.20 (6)	C8—C9—H9A	120.1
C13^i^—Fe1—C15^i^	68.81 (6)	C10—C9—H9A	120.1
C13—Fe1—C15^i^	107.13 (6)	O2—C10—C11	124.73 (14)
C15—Fe1—C15^i^	165.06 (9)	O2—C10—C9	115.18 (14)
C14—Fe1—C17	68.53 (7)	C11—C10—C9	120.09 (14)
C14^i^—Fe1—C17	150.90 (7)	C10—C11—C12	119.47 (14)
C13^i^—Fe1—C17	167.79 (8)	C10—C11—H11A	120.3
C13—Fe1—C17	40.43 (8)	C12—C11—H11A	120.3
C15—Fe1—C17	68.65 (6)	C11—C12—C7	121.31 (14)
C15^i^—Fe1—C17	118.24 (6)	C11—C12—H12A	119.3
C14—Fe1—C17^i^	150.90 (7)	C7—C12—H12A	119.3
C14^i^—Fe1—C17^i^	68.53 (7)	C17—C13—C14	108.14 (15)
C13^i^—Fe1—C17^i^	40.43 (8)	C17—C13—Fe1	70.00 (10)
C13—Fe1—C17^i^	167.79 (8)	C14—C13—Fe1	68.85 (9)
C15—Fe1—C17^i^	118.24 (6)	C17—C13—H13A	125.9
C15^i^—Fe1—C17^i^	68.64 (6)	C14—C13—H13A	125.9
C17—Fe1—C17^i^	130.42 (11)	Fe1—C13—H13A	125.9
C14—Fe1—C16^i^	165.87 (6)	C13—C14—C15	108.36 (15)
C14^i^—Fe1—C16^i^	68.74 (7)	C13—C14—Fe1	70.35 (9)
C13^i^—Fe1—C16^i^	68.23 (7)	C15—C14—Fe1	70.25 (9)
C13—Fe1—C16^i^	129.00 (7)	C13—C14—H14A	125.8
C15—Fe1—C16^i^	152.47 (6)	C15—C14—H14A	125.8
C15^i^—Fe1—C16^i^	40.85 (6)	Fe1—C14—H14A	125.8
C17—Fe1—C16^i^	109.86 (7)	C16—C15—C14	106.99 (14)
C17^i^—Fe1—C16^i^	40.52 (7)	C16—C15—P1	128.17 (12)
C14—Fe1—C16	68.74 (7)	C14—C15—P1	124.65 (12)
C14^i^—Fe1—C16	165.87 (6)	C16—C15—Fe1	69.78 (8)
C13^i^—Fe1—C16	129.01 (7)	C14—C15—Fe1	68.54 (8)
C13—Fe1—C16	68.23 (7)	P1—C15—Fe1	122.87 (8)
C15—Fe1—C16	40.85 (6)	C17—C16—C15	108.23 (15)
C15^i^—Fe1—C16	152.47 (6)	C17—C16—Fe1	69.63 (9)
C17—Fe1—C16	40.52 (7)	C15—C16—Fe1	69.37 (8)
C17^i^—Fe1—C16	109.86 (7)	C17—C16—H16A	125.9
C16^i^—Fe1—C16	119.82 (9)	C15—C16—H16A	125.9
C15—P1—C7	100.31 (7)	Fe1—C16—H16A	125.9
C15—P1—C1	102.46 (7)	C13—C17—C16	108.27 (15)
C7—P1—C1	103.32 (7)	C13—C17—Fe1	69.57 (9)
C4—O1—C19	118.61 (14)	C16—C17—Fe1	69.85 (9)
C10—O2—C18	117.95 (13)	C13—C17—H17A	125.9
C6—C1—C2	117.43 (14)	C16—C17—H17A	125.9
C6—C1—P1	120.27 (11)	Fe1—C17—H17A	125.9
C2—C1—P1	121.37 (12)	O2—C18—H18A	109.5
C3—C2—C1	121.15 (15)	O2—C18—H18B	109.5
C3—C2—H2A	119.4	H18A—C18—H18B	109.5
C1—C2—H2A	119.4	O2—C18—H18C	109.5
C2—C3—C4	120.68 (16)	H18A—C18—H18C	109.5
C2—C3—H3A	119.7	H18B—C18—H18C	109.5
C4—C3—H3A	119.7	O1—C19—H19A	109.5
O1—C4—C3	115.40 (15)	O1—C19—H19B	109.5
O1—C4—C5	125.18 (15)	H19A—C19—H19B	109.5
C3—C4—C5	119.41 (15)	O1—C19—H19C	109.5
C4—C5—C6	119.23 (14)	H19A—C19—H19C	109.5
C4—C5—H5A	120.4	H19B—C19—H19C	109.5
			
C15—P1—C1—C6	−158.86 (12)	C1—P1—C15—C16	106.83 (14)
C7—P1—C1—C6	−54.93 (14)	C7—P1—C15—C14	174.94 (13)
C15—P1—C1—C2	32.47 (15)	C1—P1—C15—C14	−78.80 (14)
C7—P1—C1—C2	136.39 (14)	C7—P1—C15—Fe1	89.69 (10)
C6—C1—C2—C3	−0.8 (3)	C1—P1—C15—Fe1	−164.05 (9)
P1—C1—C2—C3	168.20 (15)	C14—Fe1—C15—C16	−118.64 (13)
C1—C2—C3—C4	−0.3 (3)	C14^i^—Fe1—C15—C16	171.34 (10)
C19—O1—C4—C3	−178.81 (19)	C13^i^—Fe1—C15—C16	130.58 (10)
C19—O1—C4—C5	2.0 (3)	C13—Fe1—C15—C16	−80.81 (10)
C2—C3—C4—O1	−177.80 (17)	C15^i^—Fe1—C15—C16	−157.63 (9)
C2—C3—C4—C5	1.4 (3)	C17—Fe1—C15—C16	−37.27 (10)
O1—C4—C5—C6	177.74 (15)	C17^i^—Fe1—C15—C16	88.20 (11)
C3—C4—C5—C6	−1.4 (2)	C16^i^—Fe1—C15—C16	55.0 (2)
C2—C1—C6—C5	0.8 (2)	C14^i^—Fe1—C15—C14	−70.02 (15)
P1—C1—C6—C5	−168.32 (12)	C13^i^—Fe1—C15—C14	−110.77 (10)
C4—C5—C6—C1	0.3 (2)	C13—Fe1—C15—C14	37.84 (10)
C15—P1—C7—C12	77.75 (14)	C15^i^—Fe1—C15—C14	−38.99 (9)
C1—P1—C7—C12	−27.83 (15)	C17—Fe1—C15—C14	81.37 (11)
C15—P1—C7—C8	−99.24 (12)	C17^i^—Fe1—C15—C14	−153.16 (10)
C1—P1—C7—C8	155.18 (12)	C16^i^—Fe1—C15—C14	173.61 (13)
C12—C7—C8—C9	−1.7 (2)	C16—Fe1—C15—C14	118.64 (13)
P1—C7—C8—C9	175.54 (12)	C14—Fe1—C15—P1	118.25 (14)
C7—C8—C9—C10	0.9 (2)	C14^i^—Fe1—C15—P1	48.23 (13)
C18—O2—C10—C11	−0.6 (2)	C13^i^—Fe1—C15—P1	7.48 (12)
C18—O2—C10—C9	179.14 (15)	C13—Fe1—C15—P1	156.09 (12)
C8—C9—C10—O2	−178.84 (14)	C15^i^—Fe1—C15—P1	79.27 (9)
C8—C9—C10—C11	0.9 (2)	C17—Fe1—C15—P1	−160.38 (12)
O2—C10—C11—C12	177.88 (14)	C17^i^—Fe1—C15—P1	−34.91 (12)
C9—C10—C11—C12	−1.9 (2)	C16^i^—Fe1—C15—P1	−68.14 (18)
C10—C11—C12—C7	1.1 (2)	C16—Fe1—C15—P1	−123.11 (14)
C8—C7—C12—C11	0.7 (2)	C14—C15—C16—C17	0.31 (18)
P1—C7—C12—C11	−176.27 (12)	P1—C15—C16—C17	175.46 (12)
C14—Fe1—C13—C17	119.74 (14)	Fe1—C15—C16—C17	58.97 (11)
C14^i^—Fe1—C13—C17	−157.13 (9)	C14—C15—C16—Fe1	−58.66 (10)
C13^i^—Fe1—C13—C17	169.20 (10)	P1—C15—C16—Fe1	116.49 (12)
C15—Fe1—C13—C17	81.54 (10)	C14—Fe1—C16—C17	−81.42 (11)
C15^i^—Fe1—C13—C17	−113.69 (10)	C14^i^—Fe1—C16—C17	−149.5 (3)
C17^i^—Fe1—C13—C17	−45.9 (4)	C13^i^—Fe1—C16—C17	171.18 (11)
C16^i^—Fe1—C13—C17	−73.95 (12)	C13—Fe1—C16—C17	−37.41 (11)
C16—Fe1—C13—C17	37.49 (10)	C15—Fe1—C16—C17	−119.76 (14)
C14^i^—Fe1—C13—C14	83.13 (13)	C15^i^—Fe1—C16—C17	47.99 (19)
C13^i^—Fe1—C13—C14	49.46 (9)	C17^i^—Fe1—C16—C17	129.68 (13)
C15—Fe1—C13—C14	−38.20 (9)	C16^i^—Fe1—C16—C17	86.11 (10)
C15^i^—Fe1—C13—C14	126.58 (10)	C14—Fe1—C16—C15	38.34 (9)
C17—Fe1—C13—C14	−119.74 (14)	C14^i^—Fe1—C16—C15	−29.7 (3)
C17^i^—Fe1—C13—C14	−165.6 (3)	C13^i^—Fe1—C16—C15	−69.06 (12)
C16^i^—Fe1—C13—C14	166.31 (10)	C13—Fe1—C16—C15	82.34 (10)
C16—Fe1—C13—C14	−82.24 (10)	C15^i^—Fe1—C16—C15	167.75 (9)
C17—C13—C14—C15	1.07 (18)	C17—Fe1—C16—C15	119.76 (14)
Fe1—C13—C14—C15	60.23 (10)	C17^i^—Fe1—C16—C15	−110.57 (10)
C17—C13—C14—Fe1	−59.16 (11)	C16^i^—Fe1—C16—C15	−154.14 (10)
C14^i^—Fe1—C14—C13	−112.69 (11)	C14—C13—C17—C16	−0.88 (19)
C13^i^—Fe1—C14—C13	−154.99 (10)	Fe1—C13—C17—C16	−59.33 (11)
C15—Fe1—C14—C13	118.92 (14)	C14—C13—C17—Fe1	58.45 (11)
C15^i^—Fe1—C14—C13	−72.72 (12)	C15—C16—C17—C13	0.34 (19)
C17—Fe1—C14—C13	37.24 (10)	Fe1—C16—C17—C13	59.15 (11)
C17^i^—Fe1—C14—C13	173.79 (14)	C15—C16—C17—Fe1	−58.81 (11)
C16^i^—Fe1—C14—C13	−48.9 (3)	C14—Fe1—C17—C13	−37.56 (10)
C16—Fe1—C14—C13	80.90 (11)	C14^i^—Fe1—C17—C13	45.70 (18)
C14^i^—Fe1—C14—C15	128.39 (10)	C13^i^—Fe1—C17—C13	−153.8 (3)
C13^i^—Fe1—C14—C15	86.09 (11)	C15—Fe1—C17—C13	−81.98 (10)
C13—Fe1—C14—C15	−118.92 (14)	C15^i^—Fe1—C17—C13	83.40 (11)
C15^i^—Fe1—C14—C15	168.36 (7)	C17^i^—Fe1—C17—C13	168.49 (10)
C17—Fe1—C14—C15	−81.68 (10)	C16^i^—Fe1—C17—C13	127.43 (10)
C17^i^—Fe1—C14—C15	54.88 (18)	C16—Fe1—C17—C13	−119.54 (14)
C16^i^—Fe1—C14—C15	−167.8 (3)	C14—Fe1—C17—C16	81.98 (11)
C16—Fe1—C14—C15	−38.02 (9)	C14^i^—Fe1—C17—C16	165.24 (13)
C13—C14—C15—C16	−0.85 (17)	C13^i^—Fe1—C17—C16	−34.3 (4)
Fe1—C14—C15—C16	59.44 (10)	C13—Fe1—C17—C16	119.54 (14)
C13—C14—C15—P1	−176.22 (11)	C15—Fe1—C17—C16	37.56 (10)
Fe1—C14—C15—P1	−115.93 (11)	C15^i^—Fe1—C17—C16	−157.06 (10)
C13—C14—C15—Fe1	−60.29 (11)	C17^i^—Fe1—C17—C16	−71.97 (10)
C7—P1—C15—C16	0.58 (15)	C16^i^—Fe1—C17—C16	−113.03 (12)

Symmetry code: (i) −*x*, *y*, −*z*+1/2.
